# Association of Chronic Heart Failure with Frailty, Malnutrition, and Sarcopenia Parameters in Older Patients—A Cross-Sectional Study in a Geriatric Ward

**DOI:** 10.3390/jcm12062305

**Published:** 2023-03-16

**Authors:** Miroslaw Charkiewicz, Zyta Beata Wojszel, Agnieszka Kasiukiewicz, Lukasz Magnuszewski, Aleksandra Wojszel

**Affiliations:** 1 Department of Cardiology, Hospital of the Ministry of Interior and Administration in Bialystok, 15-471 Bialystok, Poland; 2 Department of Geriatrics, Medical University of Bialystok, 15-471 Bialystok, Poland; 3 Department of Geriatrics, Hospital of the Ministry of Interior and Administration in Bialystok, 15-471 Bialystok, Poland; 4 Doctoral Studies, Faculty of Health Sciences, Medical University of Bialystok, 15-471 Bialystok, Poland; 5 Interdisciplinary Student’s Scientific Society at the Department of Geriatrics, Faculty of Health Sciences, Medical University of Bialystok, 15-471 Bialystok, Poland

**Keywords:** heart failure, sarcopenia, frailty, malnutrition, older adults, comprehensive geriatric assessment, multimorbidity

## Abstract

The need to assess sarcopenia and frailty in patients with chronic heart failure (HF) has recently been raised. This cross-sectional study of 416 geriatric ward patients (median age (Me)—82 (IQR, 77–86) years, 77.4% female, 96.9% community dwelling) aimed to assess the prevalence of dynapenia, frailty syndrome, functional and nutritional health, and co-morbidity regarding their HF status. We collected data from comprehensive geriatric assessment. We observed HF in 162 (38.9%) patients, with 80 (49.4%) classified as New York Heart Association (NYHA) class III or IV. HF patients were significantly older, more frequently male, obese, hospitalized in the previous year, burdened with multimorbidity and polypharmacy, classified as frail, dependent on daily living activities, and physically non-active. Ischemic heart disease, atrial fibrillation, diabetes, peripheral arterial disease, anemia, chronic kidney disease, history of myocardial infarction, and stroke were found significantly more often in the HF group. A considerably higher percentage of HF patients had dynapenia (54.9% versus 41.9%, *p* = 0.02), but the difference was significant only in women. We found no significant difference between HF and no-HF groups regarding muscle performance, except for lower median gait speed in the HF group—0.53 m/s (0.35–0.89 m/s) versus 0.68 m/s (0.44–0.99 m/s), *p* = 0.02). HF patients significantly more often had low grip strength accompanied by slow gait, suggesting probable severe sarcopenia (40.4% vs. 29% in patients without HF, *p* = 0.046). In the regression analysis, significantly higher odds for HF were observed for lower mid-arm circumference (MAC) and dynapenia when controlling for age, sex, body mass index (BMI), calf circumference (CC), peripheral arterial disease, history of stroke, ischemic heart disease, atrial fibrillation, and diabetes mellitus. Conclusions: HF geriatric patients are often burdened with frailty, obesity, multimorbidity, and polypharmacy. As a result, they are more likely to present low muscle strength (potential sarcopenia), which is frequently accompanied by functional limitations (suggestive of more advanced stages of sarcopenia). This tendency is evident mainly in older women. Nevertheless, sarcopenia can be independently associated with HF in older patients with multimorbidity and disability who are hospitalized in a geriatric department, as a multivariable logistic regression analysis demonstrated.

## 1. Introduction

The number of patients with heart failure (HF) is growing worldwide, mainly due to the aging of the population and the increasing prevalence of risk factors for cardiovascular diseases (i.e., obesity, diabetes, hypertension). Epidemiological data suggest that HF can affect about 1–2% of the adult population. In subsequent cohorts of adulthood, this percentage increases, and in the population over 70 years of age, the prevalence of HF is already 10% [1]. Better treatment of the diseases underlying heart failure contributes to the decline in the standardized incidence of heart failure in developed countries. The first symptoms of HF are now observed later in more advanced-age patients with a greater degree of multimorbidity. 

Nevertheless, the burden of HF is rising [2]. The increased survival of patients with HF, resulting from the modern, more effective, and evidence-based scientific treatment of underlying diseases, can also influence this outcome. In addition, socioeconomically deprived people appear to be more likely to develop heart failure than wealthy people, which points to the potentially avoidable nature of heart failure that still needs to be addressed [3].

Heart failure is the cause of frequent hospitalizations and mortality [1]. In studies carried out under the European HF registry, the prognosis of patients after one year of follow-up deteriorated with the degree of reduction in the left ventricular ejection fraction (EF) and age advancement. Moreover, patients in the HF class III/IV—according to the New York Heart Association (NYHA)—the group with chronic kidney disease, low systolic blood pressure, high heart rate, or atrial fibrillation (in patients with preserved EF) had a worse prognosis. Regardless of EF, lower body mass index (BMI) was an independent factor associated with mortality in patients with HF [4]. Epidemiological data suggest that the disease is frequently associated with the involuntary loss of body weight and muscle wasting, which can determine the course of the disease and its prognosis. Therefore, in recent years, the need to assess the occurrence of sarcopenia, frailty syndrome, and malnutrition as factors negatively affecting treatment outcomes and patients’ quality of life in chronic HF has been raised [5,6,7]. This could create an opportunity for a therapeutic intervention affecting the prognosis of patients with HF [8].

The prevalence of sarcopenia in HF varies due to differences between study populations. However, it is the highest among those hospitalized for HF [9]. Nevertheless, there is a lack of research on this topic among patients in geriatric wards, who are highly disabled and burdened with multiple diseases. Therefore, this study aimed to evaluate health, nutritional, sarcopenia, and frailty parameters associated with HF in geriatric ward inpatients. Furthermore, as older patients with chronic HF may develop sarcopenia before losing weight and becoming cachectic [10], we hypothesized an independent relationship between heart failure and dynapenia (possibly sarcopenia) while controlling for the influence of other nutritional and health status parameters.

## 2. Materials and Methods

### 2.1. Patients’ Characteristics

We performed a cross-sectional study of patients consecutively admitted to the geriatric ward of the Hospital of the Ministry of Interior in Bialystok, Poland between 1st September 2014 and 30th April 2015. Based on the comprehensive geriatric assessment (CGA)—a routine interdisciplinary procedure applied in the department—we collected information on the sociodemographic, medical, nutritional, and functional characteristics of patients with (HF+) and without (HF−) heart failure. The CGA is carried out by the ward’s doctors, nurses, the physiotherapist, and the psychologist using various assessment instruments and tests designed to assess these domains, as described below, to allow for a multidomain intervention and improve outcomes [11]. We classified patients as HF+ based on a prior diagnosis or, in newly diagnosed cases, on clinical symptoms present on admission (according to the New York Heart Association Classification) and the results of transthoracic echocardiography (TTE).

Sociodemographic characteristics included age, gender, place of residence (urban/rural), and way of living (alone/with others). Medical features included information on 15 chronic diseases (as listed in the footnote to Table 1), the number of medications taken at admittance (and detailed information on drugs used in cardiovascular diseases), and hospitalizations in the last 12 months. We defined polypharmacy as five or more drugs taken and multimorbidity as five or more diseases of the 15 listed above. We collected data on renal function, the prevalence of anemia (hemoglobin below 8.69 mmol/L in men and below 7.45 mmol/L in women), thromboembolic risk (a CHA_2_DS_2_-VASc scale score > 3), and risk of bleeding (a HAS-BLED scale score ≥ 3).

We assessed blood pressure and pulse pressure at admittance and defined high pulse pressure as the difference between systolic and diastolic blood pressure above 50 mmHg. Orthostatic hypotension was diagnosed by a physiotherapist in the Active Standing Test as a drop in blood pressure of at least 20 mmHg for systolic blood pressure and at least 10 mmHg for diastolic blood pressure within 3 min of standing up. 

Information on nutritional status included data on the risk of malnutrition with Mini Nutritional Assessment-Short Form (MNA-SF) [12]; BMI; waist circumference; waist–hip ratio (WHR); calf circumference (CC); mid-arm circumference (MAC); and albumin level. Obesity was diagnosed if BMI > 30 kg/m^2^. Abdominal obesity was diagnosed if a waist circumference was >80 cm in females and >94 cm in men; it was considered second grade if a waist circumference was >88 cm in females and >102 cm in men. Patients were classified as at nutritional risk if their MNA-SF score was <12 points and as malnourished if it was <8. Low muscle mass was suspected if MAC was ≤22 cm or CC was ≤31 cm [12,13].

Slowness and weakness were diagnosed according to the criteria proposed in the literature. Cut-off points were stratified by gender and height in the case of slowness and by gender and BMI quartiles in the case of weakness [14]. The diagnosis of dementia at discharge was based on a thorough neuropsychological examination.

Frailty was diagnosed with the seven-item Canadian Study of Health and Aging Clinical Frailty Scale (CFS). Patients were classified as frail if they belonged to category 5–7, pre-frail—category 4, and robust—category 1–3 score [15]. The hand grip strength (HGS) of the dominant hand (mean of two measurements) was assessed using a manual hydraulic dynamometer SAEHAN DHD-1 [16]. Dynapenia (or probable sarcopenia) was diagnosed in men if HGS was lower than 27 kg and in women if it was lower than 16 kg. Gait speed was measured during the 4.57 m walk at the usual pace and was classified as slow when gait speed ≤0.8 m/s; low performance was measured with the Timed Up and Go test (TUG) test and diagnosed if the test result was ≥20 s [17]. For the self-reported level of physical activity, the four-level Saltin–Grimby Physical Activity Level Scale (SGPALS) was used [18]. Patients were classified as physically inactive if, during leisure time, they were mainly reading, watching television, using computers, or doing other sedentary activities.

We assessed the physical and mental abilities of an older person based on the results of the routine comprehensive geriatric assessment carried out in the geriatric ward: the ability to perform basic activities of daily life (with the Barthel Index), instrumental activities of daily living (with six items of the Duke Older American Resources and Services (OARS) I-ADL(IADL)) [19], the risk of recurrent falls (with the Performance-Oriented Mobility Assessment (POMA) [20] and TUG [21]), the risk of pressure sores (with the Norton Scale), cognitive abilities (with the Abbreviated Mental Test Score (AMTS) [21,22]), and the emotional state (with the 15-item Geriatric Depression Scale (GDS) [23]). 

### 2.2. Statistical Analysis

The IBM SPSS Version 18 Software suite (SPSS, Chicago, IL, USA) was used to analyze the data collected. The Shapiro–Wilk test was used to assess the normality of the distribution of the quantitative variables. Descriptive statistics for continuous variables were expressed as mean (M) and standard deviation (SD) or median (Me) and interquartile range (IQR) as appropriate. Categorical variables were expressed as frequency (N) and percentage (%). As appropriate, differences between groups were expressed using χ^2^ or Fisher’s exact test, the Mann–Whitney test, or the Student’s t-test. Missing values were omitted, and statistics were calculated for the adequately reduced groups. We performed a multivariable logistic regression analysis to determine the association between nutritional and sarcopenia predictors with HF, including predictors with a *p*-value less than 0.1, excluding those highly correlated (to avoid multicollinearity) and controlling for the influence of age, gender, and a number of chronic diseases. We reported odd ratios (ORs) with 95% confidence intervals (Cis) and *p* values for each model parameter. Finally, we evaluated the statistical significance of the model with the Hosmer–Lemeshow goodness-of-fit C-statistics (significant *p*-value indicating an overall lack of fit). The results were considered statistically significant at two-tailed *p* < 0.05. The computed minimum number of a sample needed to have a confidence level of 95% with a real value of within ±5% of the measured/surveyed value of 385. The calculated margin of error here was 4.68% [24].

### 2.3. Ethics Approval

The Ethics Committee approved the source study at the Medical University of Bialystok. All procedures performed in the study were under the ethical standards of the Medical University of Bialystok research committee and with the Helsinki declaration. The study can be classified as a study of ‘usual practice’. All study participants gave their informed written consent to participate in it.

## 3. Results

### 3.1. Study Cohort Characteristics—Sociodemographic and Health Correlates of Heart Failure

**Figure 1:**
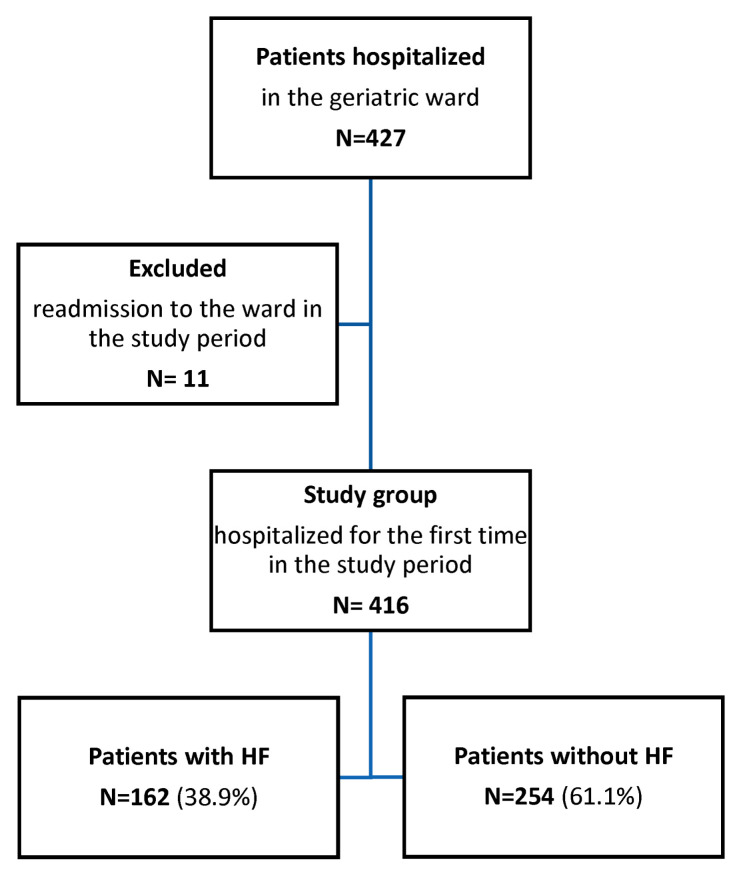
Flow chart of patient enrollment.

**Figure 2:**
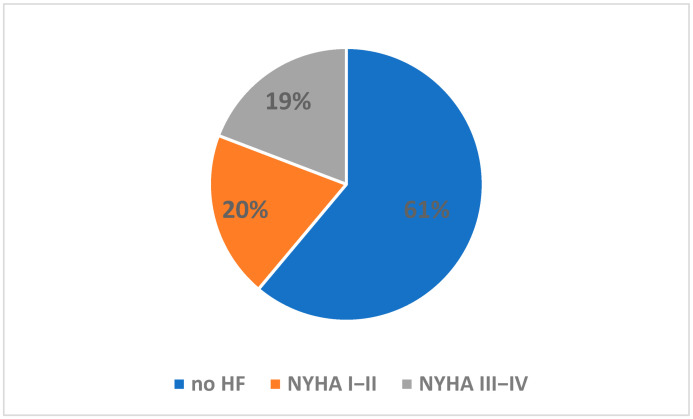
Prevalence of heart failure (HF) in the study group, where: NYHA, New York Heart Association.

Figure 1 shows patient enrollment in the study. Our research showed that out of 416 people hospitalized in the geriatric ward enrolled in the study, 162 (38.9%) had chronic heart failure (Figure 2). 

A total of 82 cases (50.6% of the HF group) were classified as NYHA class I or II and 80 cases (49.4%) as NYHA class III or IV. TTE examination was performed in only 35 patients (21.9% of the HF group) during their hospital stay. In addition, 35% of HF patients presented with swelling/peripheral edema, and 29.4% with pulmonary crepitation (in 23 cases (14.2%), both symptoms were present).

Table 1 presents the characteristics of the study group. HF patients were significantly older (Me—83, IQR 78–87 years vs. Me—82, IQR 76–85 years in the non-HF group, *p* = 0.001) and more frequently belonged to the group of the more advanced age—75+ years old (90.7% versus 79.9%, *p* = 0.004). In addition, more often, they were male (27.8% versus 19.3%; *p* = 0.05) and less frequently lived alone. 

HF+ patients were significantly more often burdened with multimorbidity (88.3% versus 37.8%, *p* < 0.001) and polypharmacy (90.0% versus 72.1%, *p* = 0.001) and had been hospitalized in the previous year (39.4% versus 23.3%, *p* = 0.001). They were more often diagnosed with ischemic heart disease, atrial fibrillation, diabetes, peripheral arterial disease, and a history of myocardial infarction and stroke. Significantly more often, they were diagnosed with anemia (although the median hemoglobin level was similar in both groups) and chronic kidney disease (substantially higher serum creatinine and lower GFR). The HF+ group could be classified as patients with an increased risk of thromboembolic complications according to the CHA2DS2-VASc score (contrary to 94.1% of the non-HF group, *p* = 0.001). Moreover, they significantly more often had a high risk of bleeding according to the HAS-BLED scale. Systolic and diastolic blood pressure in the HF+ group was considerably lower at admittance, and so was pulse pressure. The prevalence of high pulse pressure was similar in both groups, and the groups did not differ in the frequency of orthostatic hypotension. The HF+ group participants significantly more often received beta-blockers, alfa1-blockers (only by men), digoxin, ACEI/ARB, oral anticoagulants, and diuretics (significantly more loop diuretics and aldosterone receptor antagonists).

### 3.2. Study Cohort Characteristics—Functional and Nutritional Correlates of Heart Failure

The study groups significantly differed in several nutritional and functional parameters (Table 2). The mean value of the BMI was significantly higher in the HF+ group. Patients with HF were more often classified as obese and—on the verge of significance—as having abdominal obesity. The median value of MNA-SF was similar in HF and non-HF groups. According to this scale, the percentage of patients at risk of malnutrition was similar in HF and non-HF groups. The mean values of CC or MAC parameters were significantly higher in the HF group, and the percentage of participants classified as low muscle mass with these parameters was similar in both groups.

The median value of handgrip strength was significantly lower in the HF+ group, and a considerably higher percentage of the HF+ group was classified as dynapenic (with probable sarcopenia) according to EWGSOP2 sarcopenia cut-off points. When analyzed in sex groups, the significant differences in these parameters were observed in women only. Weakness was diagnosed more frequently than low grip strength and was observed significantly more often in the HF+ group.

The median value of gait speed was significantly lower in HF patients, but HF+ and HF- groups did not differ in the percentage of participants with a slow gait. Significantly more often, HF+ patients were classified as frail and severely frail, had higher POMA and TUG scores, and were more often classified as at falling risk. They had lower scores on the Barthel Index, the Duke OARS IADL scale, and the Norton scale, more often had pressure sores at admission and were physically non-active.

### 3.3. Heart Failure, Sarcopenia, and Severe Sarcopenia

We divided people assessed for muscle strength (based on hand grip strength) and fitness (using TUG or walking speed) into four groups depending on the results of these tests; see Figure 2.

**Figure 3:**
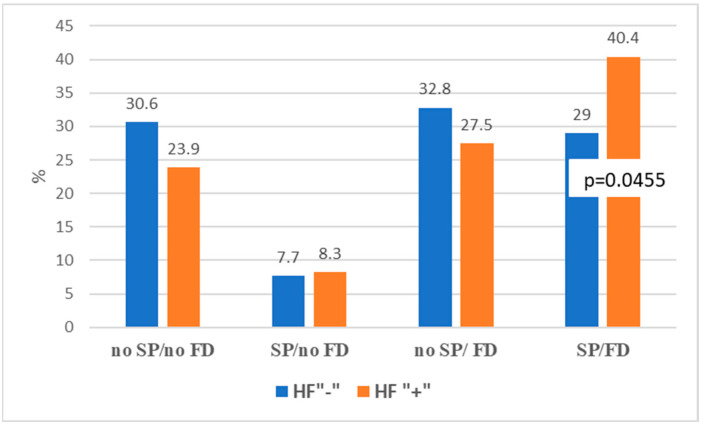
Co-prevalence of sarcopenia (SP) and functional decline (FD) in heart failure (HF“+”) and non-heart failure (HF“−“) study group, where: SP—probable sarcopenia (low hand grip strength according to EWGSOP2); FD—functional decline (gait speed ≤ 0.8 m/s and/or TUG ≥ 20 s).

In the first group, there were people categorized as fit and not suspected of sarcopenia—they did not have reduced muscle strength (no sarcopenia (SP) and no functional decline (FD)). In the second group, there were patients with reduced muscle strength but with the correct gait speed (SP but no FD); this was the smallest group of respondents. The third group included patients with a slow walking speed or a prolonged TUG time but with preserved muscle strength (no SP but FD). The fourth group included people with low grip strength accompanied by slow gait (SP and FD). The last group characteristic may be treated as the equivalent of ‘probable severe sarcopenia’. Patients with HF—Figure 3—belonged to this group significantly more often than participants without HF (40.4% vs. 29%, *p* = 0.046).

### 3.4. Heart Failure and Its Determinants—A Multivariable Logistic Regression Analysis

In the first step, we performed a multivariable logistic regression analysis on HF as a dependent variable and a block of nine independent predictors: age, sex, MNA-SF score, gait speed, potential sarcopenia (dynapenia), MAC, CC, BMI, waist circumference, as shown in Table 3 (Model 1; the overall success rate for the model was 68.7%, with a sensitivity of 46.6% and a specificity of 83%; Nagelkerk’s R2 = 0.208). In a backward analysis, six of these variables, but not MNA-SF, gait speed, and waist circumference, were retained; and age, dynapenia, CC, and BMI were significant variables (Model 2; the overall success rate for the model was 67.2, with a specificity of 82.4% and a 43.7% sensitivity; Nagelkerk’s R^2^ = 0.200). In the next step, we added the number of chronic diseases (of 15 tested) to Model 2. The regression analysis confirmed the significance of three variables: CC, MAC, and the number of chronic diseases. The overall success rate for Model 3 was 77.5%, with a specificity of 74.8% and a sensitivity of 74.8% (Nagelkerk’s R^2^ = 0.421). The Hosmer–Lemeshow goodness-of-fit *p*-values for Models 1–3 were above 0.05, leading to the rejection of the null hypothesis of a lack of fit.

## 4. Discussion

The study aimed to evaluate the prevalence of HF and its functional and nutritional correlates in geriatric ward patients, emphasizing parameters that may indicate sarcopenia.

Our results confirmed HF as one of the most frequent conditions in this group; it was present in 38.9% of patients admitted to the geriatrics department. This appears to be a very high prevalence rate. In French studies on HF in people over 80 in geriatric facilities, the frequency of HF findings was lower—about 20%. Still, these studies included cases of patients with typical exacerbation symptoms or subjects hospitalized due to exacerbations of HF [25]. We also confirmed several factors of increased risk of HF occurrence: patients with this diagnosis were significantly older, more often male, and burdened with of more accompanying chronic diseases. In addition, they were significantly more often hospitalized during the last 12 months. Among chronic conditions included in the analysis, ischemic heart disease (including previous myocardial infarction), peripheral arteriosclerosis, prior stroke/TIA, diabetes, and chronic kidney disease (including its advanced stage with GFR <30 mL/min/1.73 m^2^) were significantly more common in people with HF, as expected. Therefore, according to some authors, HF is one of the geriatric syndromes and a common pathophysiological pathway for several diseases [26]. Co-occurring chronic diseases determined a naturally high risk of thromboembolic complications in this group, accompanied significantly more often than in patients without HF, with an increased risk of bleeding complications. HF was more often accompanied by anemia, which may also result from the more frequent use of oral anticoagulants in this group due to the significantly more frequent occurrence of atrial fibrillation. However, it is worth noting that out of 96 people with atrial fibrillation, only 64 were on NOAC or VKA. This may result from either the doctor’s decision or the patient’s choice [27]. In addition, we carried out the research at a time when low-dose aspirin was considered an alternative.

There were no significant differences in the prevalence of hypertension in our study, which may be due to the generally high prevalence of this disease in geriatric patients—hypertension was found in 78.6% of the study group. Moreover, systolic and diastolic blood pressure and pulse pressure on admission to the geriatrics department were significantly lower among people with HF than those without HF. This may result from the essence of the disease but also from more intensive therapy with drugs used in HF, which lowers blood pressure at the same time. People with HF were significantly more likely to take diuretics, ACEI / ARB, alpha1-blockers, and beta-blockers. This has also been described by other authors and results from the standards of therapeutic management in HF [25].

The group of patients with HF showed a significantly more frequent occurrence of obesity diagnosed by BMI. This may indicate that sarcopenic obesity may be a more frequent clinical problem than sarcopenia among geriatric patients with HF [28]. Malnutrition significantly worsens the quality of life and the prognosis of HF patients [29]. Although the prevalence of the risk of malnutrition and malnutrition assessed with MNA-SF was very high among our study participants, the HF+ and the HF− groups did not differ in this regard. Though weight loss is the defining element of cachexia, sarcopenia (i.e., age-related loss of muscle mass and strength/function) is not necessarily associated with changes in body weight, as a proportional increase in adipose tissue may mask declining muscle mass. From a clinical point of view, distinguishing between sarcopenia and cachexia-associated muscle wasting is virtually impossible in the advanced stages of HF. However, it should be noted that as HF progresses, muscle loss occurs earlier than fat loss. Thus, older patients with HF will develop sarcopenia first, followed by weight loss and cachexia [10].

In regression models, with the controlled effects of age, sex, MNA-SF, walking speed, MAC, CC, BMI, and waist circumference, the association of sarcopenia (defined by dynapenia) with HF was statistically significant (Models 1 and 2). When chronic diseases that correlate significantly with HF (peripheral arterial disease, history of stroke/TIA, ischaemic heart disease, atrial fibrillation, and diabetes mellitus) were included in the regression model (Model 3), dynapenia and the somatometric parameters—BMI, CC and MAC—retained their significance. The chance of HF occurrence increased with the prevalence of the probable sarcopenia (dynapenia): the higher the CC, the smaller the MAC, the latter being a more reliable indicator of lower muscle mass in HF than CC. In the group of people with edema on admission, the average CC value was significantly higher in our study (data not presented). In the absence of edema, CC and MAC may indicate low muscle mass. However, anthropometric measurements are not recommended in diagnosing sarcopenia because of many confounders as age-related changes in fat distribution. EGWSOP2 guidelines recommend BIA or DEXA, but these tests are unavailable in many medical settings. On the other hand, studies have shown their association with physical performance and poor nutritional status, and GLIM criteria for the diagnosis of malnutrition retain the possibility of using these measurements for low muscle mass if more reliable tests are unavailable [30]. We used cut-off points recommended in the period of study preparation, and one of the latest studies shows that higher cut-off points may correspond better with BIA results [31].

Frailty syndrome is a multi-dimensional, dynamic state in which a person becomes more susceptible to external and internal stressors. It results from the dysregulation and decreased efficiency of many systems, which limits the ability to maintain the body’s homeostasis and response to stress. The effect of frailty syndrome is an increased risk of disability, falls, institutionalization, hospitalization, and death. It is phenotypically manifested by weight loss, muscle weakness, slower gait, low physical activity, and mental exhaustion [14]. Heart failure and frailty are two different but related conditions. Patients with HF are six times more likely to develop frailty syndrome, and frail patients have a higher risk of developing HF [32,33]. According to a recent meta-analysis, frailty syndrome is more common in patients with HF than in the general population. It may affect up to 45% of patients with heart failure [34]. Our research also confirms this. However, in the case of patients on the geriatric ward, among whom we performed our analysis, frailty syndrome occurred in 48.8% of HF patients and as many as 65.4% of HF + patients, *p* = 0.001. This is undoubtedly due to the age structure of the respondents but also to the specificity of their multiple diseases. The assessment of frailty occurrence in patients with HF is crucial, because the condition is associated with worse outcomes and poorer treatment tolerance [35,36]. 

Sarcopenia is defined as the loss of skeletal muscle mass and the weakening of the muscles’ function (muscle strength and, consequently, physical fitness) [17]. The incidence of sarcopenia is physiologically related to age but is accelerated by chronic diseases such as cancer and HF. Sarcopenia occurs in 20% to 50% of HF patients with reduced ejection fraction, often coexists with frailty syndrome, and is associated with increased morbidity and mortality. It turned out that the loss of muscle mass had a more significant impact than the loss of body mass (and a decrease in BMI) on the deterioration of physical performance and quality of life in patients with CHF [37,38]. In a recent systematic review and meta-analysis, the pooled prevalence of sarcopenia in HF was 34% (10–69%, depending on the study). Sarcopenia prevalence for hospitalized patients was higher than for ambulatory patients, but there was no significant heterogeneity between subgroups by sex or the method used to define sarcopenia [9]. In our study, the most numerous group of patients with HF were people whose suspicion of sarcopenia (based on reduced grip strength) was accompanied by a functional decline (slowed gait or extended TUG test time). People with such characteristics can be treated as those with a high probability of severe sarcopenia, negatively affecting their fitness. Severe sarcopenia was significantly more frequent in the HF+ group than in the HF- group. 

Some authors distinguish a ‘cardio-skeletal myopathy’ that develops in HF and ‘sarcopenia’ as a derivative of the aging process. The former consists of muscle fiber and capillary atrophy, conversion of type I fibers into type II ones, change in muscle structure and fiber orientation due to intra-fibular edema, and deposition of connective and adipose tissue—which consequently impairs the ability to generate strength and exercise tolerance. The latter is a result of selective denervation, the loss of fast motor units (with type II fibers being more susceptible to atrophy than type I fibers), and fat and connective tissue infiltration contributing to the decline in muscle quality. The frequent comorbidity of sarcopenia and HF may be explained by their common pathophysiological pathways, including altered nutrient intake and absorption (malnutrition), inflammatory processes and metabolic and autonomic disorders, humoral factors, the ubiquitin-proteasome system (UPS), myostatin signaling, apoptosis, and oxidative stress. These overlapping processes result in ultrastructural muscle abnormalities, mitochondrial structure and function changes, increased oxidative stress, and a shift in fiber distribution, ultimately leading to decreased exercise capacity [10].

Our research confirmed the complexity of the clinical picture of HF in geriatric patients, indicating the fact that it is a multi-organ disease and not just a hemodynamic disorder. This means that the approach to diagnostics and therapy of HF in patients of advanced age should be multidirectional, as indicated by other authors [39]. Considering the possibility of mutual influence, it is necessary to prevent frailty syndrome and sarcopenia in old age, but if these syndromes occur, they must be treated properly. The most effective treatment strategy for sarcopenia is aerobic and resistance training combined with an appropriate supply of protein (1–1.5 g/kg/24 h) [40].

The main limitation of this study was that it surveyed only one geriatric ward; therefore, the results cannot be generalized to the entire older adult population. In addition, we collected some information retrospectively from patients’ medical records after their discharge, resulting in missing data. Moreover, we can only talk about ‘potential sarcopenia’, because we rely on the results of the assessment of muscle strength while making a diagnosis. To confirm sarcopenia, we should assess the muscle mass. However, we did not perform this assessment in our research because of the need for the required measuring equipment. On the other hand, as confirmed recently by Blanquet et al., hand grip strength can be a valuable tool to screen for sarcopenia in older patients with chronic HF [41]. Unfortunately, we could not include information on NT-proBNP, which is a good indicator to help distinguish whether a patient has heart failure. At the time of data collection, the European guidelines for managing heart failure recommended considering only the determination of natriuretic peptide levels. Therefore, our hospital ordered NT-proBNP rarely, only in selected clinical situations.

Significantly higher odds for HF are observed for lower MAC (pointing to lower muscle mass) and for probable sarcopenia defined as dynapenia when controlling for age, sex, BMI, CC, and chronic diseases correlating with HF prevalence (peripheral arterial disease, history of stroke/TIA, ischemic heart disease, atrial fibrillation, and diabetes mellitus). This may confirm that sarcopenia (defined as a loss of muscle mass and muscle strength) is an independent predictor of heart failure in older patients with multimorbidity and disability who are hospitalized in a geriatric department.

## 5. Conclusions

HF geriatric patients are often burdened with frailty, obesity, multimorbidity, and polypharmacy. As a result, they are more likely to present low muscle strength (potential sarcopenia), which is frequently accompanied by functional limitations (suggestive of more advanced stages of sarcopenia). This tendency is evident mainly in older women. Nevertheless, sarcopenia can be independently associated with HF in older patients with multimorbidity and disability who are hospitalized in a geriatric department, as a multivariable logistic regression analysis has demonstrated.

The study describes the prevalence and characteristics of HF among typical geriatric patients in a hospital ward (without excluded cases) in daily practice. HF occurred more frequently than in other studies on older populations, and the patients were burdened with numerous comorbidities and functional disabilities. The complex clinical picture of heart failure in geriatric patients confirms the need for a multidirectional and multidisciplinary approach that would correct hemodynamic disorders and prevent frailty, malnutrition, and sarcopenia.

The clinical implications of this study are as follows:

- The study confirms the high occurrence of geriatric syndromes, such as dynapenia, inactivity, and frailty, in the HF group, indicating a need for comprehensive geriatric assessment in HF patients.
- Obesity and sarcopenic obesity seem to be more frequent clinical problems than sarcopenia alone among geriatric patients with HF.
- High levels of inactivity and dynapenia in geriatric HF patients may indicate a direction of prevention and therapeutic strategies.

## Figures and Tables

**Table 1:** Patients’ characteristics—sociodemographic and health parameters.

Parameter	Total	HF + Group	HF− Group	*p* Values ^a^	Missing Data
No. (%) of patients	416 (100.0)	162 (38.9)	254 (61.1)		
Age, y, Me (IQR)	82 (77.0–86.0)	83 (78.0–87.0)	82 (76.0–85.0)	0.001	-
Age, 75+, n (%)	350 (84.1)	147 (90.7)	203 (79.9)	0.004	-
Sex, men, n (%)	94 (22.6)	45 (27.8)	49 (19.3)	0.05	-
Place of residence, rural, n (%)	87 (20.9)	36 (22.2)	51 (20.1)	0.62	
Living alone, n (%)	119 (29.8)	36 (23.5)	83 (33.7)	0.03	17
Number of chronic diseases ^b^, Me (IQR)	5.0 (3.0–6.0)	6.0 (5.0–7.0)	4.0 (3.0–5.0)	<0.001	-
Multimorbidity ^c^, n (%)	239 (57.5)	143 (88.3)	96 (37.8)	<0.001	-
Number of drugs, Me (IQR)	7.0 (5.0–9.0)	8.0 (6.0–10.0)	6.0 (4.0–9.0)	<0.001	9
Polypharmacy ^d^, n (%)	322 (79.1)	144 (90.0)	178 (72.1)	0.001	9
Hospitalization in the last year, n (%)	122 (29.5)	63 (39.4)	59 (23.3)	0.001	3
AMTS, Me (IQR)	8.0 (6.0–9.0)	8.0 (6.0–9.0)	8.0 (6.0–9.0)	0.54	35
Dementia, n (%)	133 (32.0)	44 (27.2)	89 (35.0)	0.11	-
GDS, Me (IQR)	7.0 (3.0–10.0)	7.0 (4.0–10.0)	6.0 (3.0–10.0)	0.47	52
Depression, n (%)	181 (56.9)	73 (59.8)	108 (55.1)	0.42	98
Hypertension, n (%)	327 (78.6)	133 (82.1)	194 (76.4)	0.18	-
Ischemic heart disease, n (%)	223 (53.6)	108 (66.7)	115 (45.3)	<0.001	-
Myocardial infarction, n (%)	39 (9.4)	32 (19.8)	7 (2.8)	<0.001	-
Atrial fibrillation, n (%)	98 (23.6)	71 (43.8)	27 (10.6)	<0.001	-
Peripheral arterial disease, n (%)	64 (15.4)	42 (25.9)	22 (8.7)	<0.001	-
Stroke/TIA, n (%)	56 (13.5)	29 (17.9)	27 (10.6)	0.04	-
Diabetes	126 (30.3)	63 (38.9)	63 (24.8)	0.003	-
Chronic osteoarthritis, n (%)	324 (77.9)	132 (81.5)	192 (75.6)	0.18	-
COPD/asthma	42 (10.1)	21 (13.0)	21 (8.3)	0.14	
Systolic BP at admittance, mmHg, Me (IQR)	130 (120–140)	125 (110–140)	130 (120–140)	<0.001	7
Diastolic BP at admittance, mmHg, Me (IQR)	70 (60–80)	70 (60–80)	70 (65–80)	0.02	7
Pulse pressure at admittance, mmHg, Me (IQR)	60 (50–65)	55 (47.25–60)	60 (50–70)	0.007	7
High pulse pressure (>50 mmHg)	248 (60.6)	88 (55.0)	160 (64.3)	0.06	7
Orthostatic hypotension, n (%)	57 (16.1)	22 (16.4)	35 (16.0)	1.0	63
Creatinine, mg/dL Me (IQR)	0.98 (0.84–1.19)	1.06 (0.9–1.34)	0.92 (0.80–1.09)	<0.001	11
GFR ^e^, ml/min/1.73 m^2^, M (SD)	58.1 (17.0)	52.4 (16.3)	61.8 (16.5)	<0.001	11
GFR < 60 ml/min/1.73 m^2^, n (%)	218 (52.4)	109 (67.3)	109 (42.9)	<0.001	11
GFR < 30 ml/min/1.73 m^2^, n (%)	21 (5.2)	14 (8.9)	7 (2.8)	0.01	11
Albumin < 35g/L, n (%)	58 (14.9)	22 (14.2)	36 (15.4)	0.77	27
HAS-BLED ≥ 3, n (%)	63 (15.2)	42 (26.1)	21 (8.3)	<0.001	-
CHA_2_DS_2_-VASc > 3, n (%)	399 (96.4)	161 (100%)	238 (94.1)	0.001	-
Total cholesterol, mmol/L, M (SD)	4.70 (1.25)	4.46 (1.25)	4.86 (1.22)	0.002	27
Triglycerides, mmol/L, Me (IQR)	11.54 (8.69–14.89)	12.34 (8.80–15.66)	10.97 (8.46–14.74)	0.14	102
Hemoglobin, g/dL, Me (IQR)	12.6 (11.5–13.7)	12.4 (11.2–13.7)	12.7 (11.7–13.7)	0.25	12
Anemia, n (%)	177 (43.8)	80 (50.3)	97 (39.6)	0.04	12
Iron, μg/dL, Me (IQR)	67 (46.0–88)	68 (41.50–83.0)	67 (51.5–96.5)	0.17	241
Statins, n (%)	142 (35.0)	60 (37.5)	82 (33.3)	0.40	10
ß-blockers, n (%)	258 (63.5)	123 (76.9)	135 (54.9)	<0.001	10
α1-blockers, n (%)	25 (6.2)	16 (10.0)	9 (3.7)	0.01	10
Calcium channel blockers, n (%)	114 (28.1)	53 (33.1)	61 (24.8)	0.07	10
Digoxin, n (%)	30 (7.4)	22 (13.8)	8 (3.3)	<0.001	10
Antiarrhythmics, n (%)	9 (2.2)	4 (2.5)	5 (2.0)	0.74	10
ACEI or ARB, n (%)	259 (63.8)	116 (72.5)	143 (58.1)	0.004	10
Cholecalciferol, n (%)	88 (21.7)	34 (21.3)	54 (22.0)	0.90	10
Oral anticoagulants (VKA or NOAC), n (%)	64 (15.4)	50 (30.9)	14 (5.5)	<0.001	10
Diuretics, n (%)	196 (48.3)	103 (64.4)	93 (37.8)	<0.001	10
Thiazides, n (%)	83 (20.4)	25 (15.6)	58 (23.6)	0.06	10
Loop diuretics, n (%)	100 (24.6)	70 (43.8)	30 (12.2)	<0.001	10
ARA (spironolactone, eplerenone), n (%)	71 (17.5)	53 (33.1)	18 (7.3)	<0.001	10

^a^—x^2^ test or Fisher’s exact test, as appropriate, for categorical variables; student’s *t*-test or Mann–Whitney test, as appropriate, for continuous or interval variables. In all analyses, a two-tailed *p*-value of less than 0.05 was regarded as significant; ^b^—of 15 chronic diseases (peripheral arterial disease, ischemic heart disease, chronic cardiac failure, hypertension, stroke, atrial fibrillation, chronic obstructive pulmonary disease, diabetes/prediabetes, neoplasm, thyroid gland disease, dementia, parkinsonism, chronic arthritis, chronic renal disease, dementia); ^c^—five or more diseases of the 15 listed above; ^d^—five or more drugs taken; ACEI—angiotensin-converting enzyme (ACE) inhibitors; AMTS—the Abbreviated Mental Test Score; ARA—aldosterone receptor antagonists; ARB—angiotensin receptor blockers; BP—blood pressure; CHA2DS2-VASc—a scale for the assessment of thromboembolic risk composed of: C, congestive heart failure (or left ventricular systolic dysfunction); H, hypertension; A2, age 75+ years; D, diabetes mellitus; S2, prior stroke, transient ischemic attack, or thromboembolism; V, vascular disease (e.g., peripheral artery disease, myocardial infarction, aortic plaque); A, age 65–74 years; Sc, sex category (i.e., female sex); COPD—chronic obstructive pulmonary disease; GDS—Geriatric Depression Scale; eGFR—glomerular filtration rate; HAS-BLED—a scale for the assessment of bleeding risk composed of: H, hypertension (>160 mmHg systolic); A, abnormal renal/ liver function; S, stroke history; B, prior bleeding or predisposition to bleeding; L, labile INR; E, elderly (age > 65); D, drugs (predisposing to bleeding—aspirin, clopidogrel, NSAIDs) and alcohol use (≥8 drinks/week); HF+—patients with heart failure; HF−—patients without heart failure; IQR—interquartile range; Me—median value; n—number of cases; NOAC—new oral anticoagulants; TIA—transient ischemic attack; VKA—vitamin K antagonists.

**Table 2:** Patients’ characteristics—nutritional and functional parameters.

Parameter	Total	HF + Group	HF− Group	*p* Values ^a^	Missing Data
No. (%) of patients	416 (100.0)	162 (38.9)	254 (61.1)		
BMI, kg/m^2^, M (SD)	29.3 (6.0)	30.7 (6.2)	28.4 (5.7)	<0.001	62
BMI < 24 kg/m^2^, n (%)	66 (18.6)	22 (16.4)	44 (20.0)	0.48	62
Obesity (BMI > 30 kg/m^2^), n (%)	148 (41.8)	72 (53.7)	76 (34.5)	0.001	62
WHR, Me (IQR)	0.91 (0.87–0.95)	0.91 (0.87–0.96)	0.91 (0.86–0.95)	0.35	63
Abdominal obesity 1st grade ^b^, n (%)	295 (81.0)	127 (80.8)	168 (77.8)	0.06	63
Abdominal obesity 2nd grade ^c^, n (%)	233 (64.0)	103 (69.6)	130 (60.2)	0.08	63
MNA-SF, Me (IQR)	12.0 (9.0–13.0)	11.0 (9.0–13.0)	12.0 (9.0–13.0)	0.78	12
MNA SF < 12 (malnutrition risk), n (%)	198 (49.0)	80 (50.3)	118 (48.2)	0.69	12
MNA SF < 8 (malnutrition), n (%)	72 (17.8)	24 (19.7)	48 (17.0)	0.60	12
MAC, cm, M (SD)	28.0 (4.0)	28.7 (4.0)	27.6 (3.9)	0.01	49
MAC ≤ 22cm, n (%)	89 (24.3)	29 (19.6)	60 (27.4)	0.11	49
CC, cm, M (SD)	34.4 (4.6)	35.6 (4.9)	33.6 (4.3)	<0.001	51
CC < 31 cm, n (%)	74 (20.3)	24 (16.4)	50 (22.8)	0.15	51
Handgrip, kg, Me (IQR)	18.15 (13.7–22.8)	16.7 (12.9–22.0)	18.9 (14.2–23.2)	0.04	66
women	16.7 (12.9–20.5)	15.2 (11.6–18.9)	18.0 (13.5–21.5)	0.002	
men	26 (21–32.3)	26.1 (20.5–32.3)	25.9 (21.0–32.4)	0.98	
Low strength ^d^, n (%)	164 (46.9)	73 (54.9)	91 (41.9)	0.02	66
women	124 (45.3)	55 (55.0)	69 (39.7)	0.02	
men	40 (52.6)	18 (54.5)	22 (51.2)	0.82	
Weakness ^e^, n (%)	233 (66.6)	101 (75.9)	132 (60.8)	0.004	66
Gait speed, m/s, Me (IQR)	0.65 (0.40–0.96)	0.53 (0.35–0.89)	0.68 (0.44–0.99)	0.02	102
Slowness ^e^, n (%)	166 (52.9)	68 (59.1)	98 (49.2)	0.10	102
Gait speed ≤ 0.8 m/s ^d^, n (%)	205 (65.3)	79 (68.7)	126 (63.3)	0.39	102
Gait speed ≤ 0.8 m/s or immobile, n (%)	238 (68.6)	97 (72.9)	141 (65.9)	0.19	
CFS, Me (IQR)	5.0 (4.0–5.0)	5.0 (4.0–6.0)	4.0 (4.0–5.0)	<0.001	-
Frailty, n (%)	230 (55.3)	106 (65.4)	124 (48.8)	0.001	-
Severe frailty ^e^, n (%)	102 (24.5)	50 (30.9)	52 (20.5)	0.02	-
Barthel Index, Me (IQR)	90.0 (70.0–100.0)	85.0 (65.0–95.0)	95.0 (70.0–100.0)	0.01	6
Duke OARS IADL, Me (IQR)	7.0 (3.0–11.0)	6.0 (2.0–9.0)	8.0 (4.0–11.0)	0.01	10
POMA, Me (IQR)	23.0 (17.0–28.0)	21.0 (16.0–28.0)	24.0 (18.0–28.0)	0.02	94
POMA < 24, n (%)	181 (56.2)	76 (63.3)	105 (52.0)	0.049	94
TUG, s, Me (IQR)	17.4 (11.87–28.0)	21.5 (12.6–52.1)	16.4 (11.5–24.1)	0.005	115
TUG ≥ 14 s, n (%)	195 (64.8)	83 (72.8)	112 (59.9)	0.03	115
TUG ≥ 20 s, n (%)	128 (42.5)	61 (53.5)	67 (35.8)	0.004	115
Norton scale, Me (IQR)	17 (15-19)	17 (15-19)	18 (15-19)	0.049	6
Pressure sores at admission, n (%)	18 (4.4)	13 (8.0)	5 (2.0)	0.005	5
Falls in the last 12 months, n (%)	157 (43.9)	66 (48.9)	91 (40.8)	0.15	58
Non-active in SGPALS, n (%)	168 (41.0)	86 (54.4)	82 (32.5)	<0.001	6

^a^—x^2^ test or Fisher’s exact test, as appropriate, for categorical variables; student’s t-test or Mann–Whitney test, as appropriate, for continuous or interval variables. A two-tailed *p*-value of less than 0.05 was considered significant in all analyses. ^b^—waist >80 cm in women and />94 cm in men; ^c^—waist >88 cm in women and >102 cm in men; ^d^—according to EWGSOP2 sarcopenia cut-off points; ^e^—according to frailty scale by Linda Fried; BMI—body mass index; CC—calf circumference; CFS—7-point Clinical Frailty Scale; IQR—interquartile range; M—mean value; Me—median value; MAC—mid-arm circumference; MNA-SF—Mini Nutritional Assessment Short Form; n—number of cases; POMA—Performance-Oriented Mobility Assessment; SGPALS—Saltin–Grimby Physical Activity Level Scale; SD—standard deviation; TUG—Timed Up and Go test; WHR—waist–hip ratio.

**Table 3:** Multivariate logistic regression models for predictors of HF.

		Model 1			Model 2			Model 3	
	OR	95% CI	*p* Value	OR	95% CI	*p* Value	OR	95% CI	*p* Value
Age	1.07	1.02–1.12	0.008	1.07	1.02–1.12	0.005	1.06	1.004–1.12	0.04
Sex (male)	2.06	0.99–4.29	0.053	1.89	0.99–3.64	0.055	1.22	0.56–2.65	0.61
MNA-SF	0.99	0.88–1.11	0.862						
Gait speed	0.78	0.30–2.06	0.621						
SP	1.87	1.03–3.40	0.041	1.97	1.11–3.50	0.021	1.94	1.01–3.73	0.045
MAC	0.90	0.79–1.02	0.106	0.89	0.79–1.01	0.077	0.83	0.72–0.96	0.012
CC	1.15	1.04–1.27	0.007	1.15	1.04–1.27	0.005	1.18	1.05–1.32	0.006
BMI	1.12	0.99–1.24	0.08	1.10	1.01–1.19	0.034	1.12	1.02–1.24	0.020
Waist circumference	0.99	0.95–1.03	0.73						
Peripheral arterial disease							1.88	0.80–4.45	0.149
History of stroke/TIA							1.89	0.72–4.98	0.195
Ischaemic heart disease							3.32	1.71–6.46	<0.001
Atrial fibrillation							9.07	4.04–20.38	<0.001
Diabetes mellitus							0.79	0.37–1.65	0.527
Negelkerk’s R^2^		0.208			0.200			0.419	
% correctly predicted		68.7%			67.2%			77.1%	
sensitivity		46.6%			43.7%			62.1%	
specificity		83.0%			82.4%			86.8%	
Hosmer–Lemeshow goodness of fit		0.799			0.447			0.208	
Regression method	Enter method			Backward analysis of Model 1			Enter method (Model 2 and diseases correlating with HF)		

BMI—body mass index; CC—calf circumference; CI—confidence interval; MAC—mid-arm circumference; MNA-SF— Mini Nutritional Assessment Short Form; OR—odds ratio; SP—probable sarcopenia (low hand grip strength according to EWGSOP2); TIA—transient ischemic attack.

## Data Availability

The data supporting the results in the current study are available from the corresponding author upon reasonable request.
